# Scoping review protocol: a mobile health solution for the management of stress among health science students at rural universities in South Africa

**DOI:** 10.11604/pamj.2026.53.156.48724

**Published:** 2026-04-14

**Authors:** Bhekithemba Vellem, Nozuko Glenrose Mangi

**Affiliations:** 1 Department of Nursing, Faculty of Medicine and Health Sciences, Walter Sisulu University, Mthatha, South Africa

**Keywords:** Mobile health, stress management, health science students, rural university

## Abstract

Stress is prevalent among health science students, particularly in rural settings where access to mental health resources is limited. Mobile health (mHealth) interventions offer a promising avenue for providing accessible support for stress management. This scoping review aims to identify existing mHealth interventions, evaluate their feasibility and acceptability, and explore the specific needs of students within these contexts. This scoping review will be conducted using the Arksey and O'Malley framework, a structured approach that consists of six stages: identifying the research question, searching for relevant studies, selecting studies, charting the data, collating and summarising the results, and consulting stakeholders. The search will include a comprehensive range of electronic databases and grey literature sources to ensure broad coverage of relevant studies. The Joanna Briggs Institute (JBI) guidelines will be used to enhance the methodology, ensuring rigour and transparency throughout the review process. A systematic search strategy will be employed across multiple databases to gather relevant literature on mHealth interventions targeting stress management among health science students. The findings of this review, which will be disseminated through academic journals and conferences, are crucial in informing policymakers, researchers, and educators about effective mHealth strategies tailored for health science students. By highlighting gaps in current research and practice, this review will contribute valuable insights that can guide future intervention development and implementation in rural settings. The findings from this review will help shape a contextually appropriate mHealth program designed to support stress management for health science students studying in rural university settings.

## Introduction

Mental health disorders are among the leading causes of disability worldwide, with stress being a significant contributor to psychological distress [1]. Health science students are at heightened risk due to the demanding nature of their academic and clinical training, often experiencing elevated levels of stress, anxiety, depression, and burnout [2]. The burden of mental health challenges is further exacerbated in low-resource settings, such as rural universities in South Africa, where students face limited access to mental health services, social isolation, and cultural adaptation difficulties in South Africa.

In response to these challenges, mobile health (mHealth) interventions have gained traction as innovative tools for improving mental health care by providing accessible, scalable, and cost-effective support mechanisms [3]. Research suggests that mHealth applications can facilitate stress management through self-guided interventions, real-time monitoring, and digital counselling, offering an alternative to traditional face-to-face mental health services, often inaccessible in rural contexts [4,5]. However, despite the growing global recognition of mHealth solutions, there remains a gap in understanding their effectiveness and implementation within the specific context of rural universities in South Africa.

This scoping review aims to systematically map the available literature on mHealth interventions for stress management among health science students in a rural university setting. This review will identify the types, effectiveness, and challenges of mHealth solutions in this demographic. The findings will contribute to the broader discourse on mental health support in low-resource academic environments and inform future strategies for integrating digital health interventions into student wellness programs.

**Strengths and limitations:** 1) the review will only include English-language studies, which may limit the findings' comprehensiveness and exclude pertinent material published in other languages; 2) the study will concentrate on research that was released between 2015 and 2025, possibly leaving out previous studies that might still provide valuable information about mHealth stress-reduction strategies; 3) the dependability of the results could be impacted by the scoping review methodology's failure to critically evaluate the calibre of the included studies; 4) the precision and comprehensiveness of the results may be constrained by the quality and accessibility of the data presented in the included research, especially if important data are either missing or presented inadequately; 5) even though several databases will be searched, there is a chance that the search technique will overlook pertinent studies.

## Methods

This scoping review will adhere to the Joanna Briggs Institute (JBI) scoping review as well as the framework described by Arksey and O'Malley's methodology [6,7]. The following steps will be part of the review. Table 1 lists the five steps that make up the review process.

**Table 1:** outlines of the planned scoping review process using the Arksey and O´Malley framework aligned with PRISMA-scoping review items to guide methodological steps for the upcoming review

Arksey and O'Malley framework	PRISMA-ScR protocol relevant checklist items
1. Identify the research questions	Objectives
2. Identifying relevant studies	Information sources, eligibility criteria, and search strategy
3. Study selection	Selection of sources for evidence purposes
4. Charting data	Process of data charting, data items, and critical appraisal
5. Collating, summarizing, and reporting findings	Summary of results

PRISMA: preferred reporting items for systematic reviews and meta-analyses; ScR: scoping reviews

**Stage 1-identifying the research questions:** a preliminary literature review was done to formulate research questions. The primary research question focuses on identifying mobile health interventions that support stress management among health science students in resource-limited settings. The following are the precise objectives of this study: 1) identify and characterize existing mHealth interventions aimed at stress management among health science students; 2) summarize evidence on the feasibility, acceptability, and user engagement of these interventions as reported in the literature; 3) explore and highlight the specific stress-related needs, challenges, and contextual factors affecting health science students in rural university settings in South Africa. The primary research question guiding this scoping review is: what mobile health interventions support stress management among health science students in resource-limited settings?

**Stage 2-identifying relevant studies:** a comprehensive search strategy will be developed in collaboration with an academic librarian. The search will include electronic databases such as PubMed, MEDLINE, Web of Science, PsycINFO, and Google Scholar. The search terms will include ‘mobile health’, ‘mHealth’, ‘stress management’, ‘health science students’, ‘rural university’, and ‘South Africa’. Grey literature will also be considered to capture relevant studies not indexed in these databases. Grey's literature sources are listed in Table 2, along with a description of how each will be searched.

**Table 2:** planned grey literature search strategy, listing data sources and commentary to support the comprehensive identification of relevant non-peer-reviewed materials

Source	Commentary
Grey literature database	GreyLit, OpenGrey via DANS EASY
Grey and published literature database	PubMed, Scopus, PsycINFO, Web of Science
Theses/dissertations	ProQuest Dissertations and Theses Global, WorldCat Dissertations and Theses
Internet search engines	Google and Google Scholar

Table 3 provides a comprehensive outline of the search strategy. The lead researcher, in conjunction with the research team and an academic librarian, will determine pertinent search terms. Keywords such as ‘mobile health’, ‘mHealth’, ‘stress management’, ‘health science students’, ‘rural university’, and ‘South Africa’ will be used and searched in titles, abstracts, and subject headings. Retrieved review articles will be screened for their titles, abstracts, and index terms. Articles from each database will be imported into Zotero for record-keeping, article tracking, and reference list creation for the final report.

**Table 3:** search strategies to be used for retrieving articles

S/N	Database	Search Term	Customization
1	PubMed	(("mobile health" or mHealth or "digital health") and ("stress management" or "mental health support")) and (("rural university" or "South Africa") and ("health science students" or "nursing students" or "medical students"))	English; Jan 2014 - Dec 2024
2	Web of Science (WOS)	(("mobile health" or mHealth or "digital intervention") and ("stress" or "mental well-being")) and (("rural context" or "South Africa") and ("undergraduate students" or "nursing students"))	English; Jan 2014 - Dec 2024
3	SCOPUS	Title-ABS-key (("mobile health" or "mHealth") and ("stress management" or "mental health intervention") and ("rural context" or "South Africa")) and ("health science students")	English; Jan 2014 - Dec 2024
4	PsycINFO	Title-ABS-key (("mobile health" or "mHealth") and ("stress management" or "mental health intervention") and ("rural context" or "South Africa")) and ("health science students")	English; Jan 2014 - Dec 2024
5	Google Scholar	("mobile health" or "mHealth" or "digital health") and ("stress" or "mental health") and ("rural university" or "South Africa") and ("health science students" or "medical students")	English; Jan 2014 - Dec 2024

**Synthesis of eligibility criteria:** peer-reviewed published papers on mobile health interventions for stress management among health science students at South African rural universities are included and excluded based on the criteria listed in Table 4: (i) English-language publications; (ii) conducted in any setting; (iii) within (specify years); (iv) using randomized controlled trials, intervention studies (including quasi-experimental designs), qualitative and quantitative methods, and (v) concentrating on health science students as the study population are all included in the inclusion criteria. Studies that don't fit these requirements won't be accepted.

**Table 4:** inclusion and exclusion criteria

S/N	Categories	Inclusion criteria	Exclusion criteria
1	Language	English studies	Non-English studies
2	Publication date range	Studies conducted from January 2015 to December 2025	Studies conducted before January 2015
3	Participant age	Health science students aged 18 and above	Participants under 18 years of age
4	Population category	Health science students at rural universities	Non-health science student populations
5	Studies	Studies focusing on mHealth interventions for stress management among health science students	Studies unrelated to stress management or without mobile health interventions
6	Study design	All primary, secondary, and peer-reviewed studies, including randomized controlled trials, qualitative and quantitative studies	Meta-analyses, systematic reviews

**Study design:** this review will consider all primary peer-reviewed study designs, including both qualitative and quantitative research. Review papers, meta-analyses, and systematic reviews, however, will not be included.

**Setting:** this review will include studies conducted in settings that focus on the development or implementation of mobile health (mHealth) solutions for stress management among health science students in rural or resource-limited university contexts.

**Stage 3-study selection:** for this scoping review, three reviewers will independently examine the titles and abstracts of identified studies to determine their eligibility. Studies will be included if they meet the following criteria: published in English between January 2014 and December 2024. Focus on mobile health interventions for stress management. The target population consists of health science students in rural universities. Any discrepancies between reviewers will be resolved through discussion or the involvement of a third-party arbitrator if needed. The selection process will be conducted in two stages:

**Title and abstract screening:** two independent reviewers will screen all retrieved citations against the inclusion criteria. Relevant meta-analyses and review articles will be excluded, but their reference lists will be scanned for potential primary studies.

**Full-text screening:** articles identified as relevant during the first stage will undergo a full-text review. The inter-rater reliability will be assessed using percentage agreement, and if the agreement is below 80%, the inclusion and exclusion criteria will be refined to ensure consistency. To enhance transparency, the Preferred Reporting Items for Systematic Reviews and Meta-Analyses (PRISMA) flowchart will be used to document the study selection process.

**Stage 4-charting the data:** a standardised data extraction form will be developed to ensure consistency in data collection. Three independent reviewers will extract data from the included studies. The data extraction form will capture the following information: author(s) and year of publication, study design and methodology, study setting (country, type of university, rural/urban classification), sample size and demographic characteristics of participants, description of mobile health intervention (features, mode of delivery, duration, and theoretical framework, if any, outcomes assessed (stress reduction, feasibility, acceptability, adherence, etc.), key findings and recommendations.

Before full implementation, a pilot test of the data extraction form will be conducted using a subset of included studies. If necessary, modifications will be made to accommodate emerging themes. Any disagreements between reviewers during data extraction will be resolved through discussion or, if necessary, mediation by a third reviewer. If crucial information is missing from eligible studies, the authors will be contacted for clarification.

**Stage 5-collating, summarising, and reporting the findings:** a narrative synthesis approach will be utilised to summarise the existing knowledge on mobile health interventions for stress management among health science students in rural universities. The data will be analysed thematically, identifying common factors such as intervention effectiveness, barriers, facilitators, and implementation challenges. Thematic analysis will be used to categorise the determinants of mHealth success, including accessibility, user engagement, technological constraints, and institutional support.

The review will also highlight gaps in the existing literature and propose directions for future research. Rather than evaluating individual study quality, this review will provide an overview of the available evidence, aggregate findings, and present a structured summary of the key themes.

**Figure 1:**
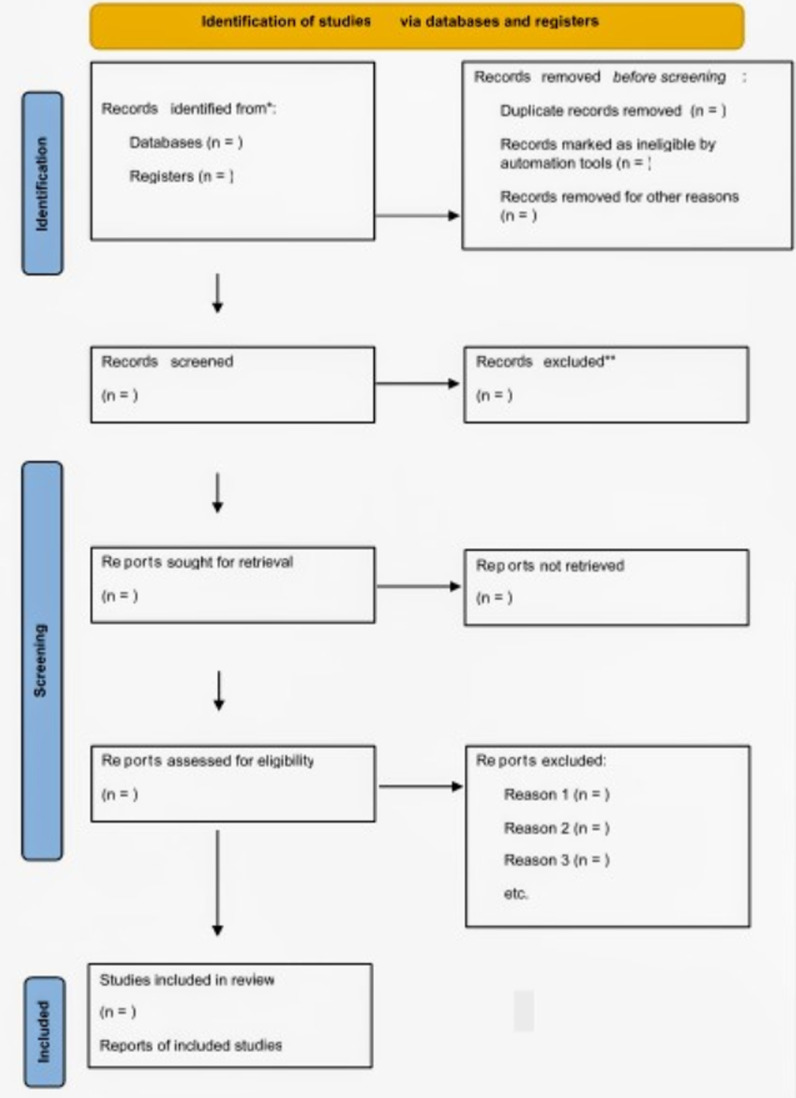
PRISMA flowchart

The findings will be disseminated through peer-reviewed publications, academic conferences, and presentations to policymakers, university administrators, and mental health practitioners. Additionally, the results will be shared with health ministries and student support organizations to inform strategies for integrating mobile health interventions into stress management solutions at rural universities. This review aims to contribute to the growing body of knowledge on digital mental health interventions and their role in improving student well-being in resource-limited settings (Figure 1) [8].

**Ethical considerations:** the study protocol received approval from the Walter Sisulu University Ethics Review Committee (NHREC Reg. No. REC-120209-020) under Ethics Approval Number 247/2024.

## Expected results

This study is expected to produce a comprehensive overview of mobile health (mHealth) interventions designed to support stress management among health science students, particularly within resource-constrained and rural university settings like South Africa. The study selection process will be shown in a PRISMA [7] flow diagram, offering a clear account of how sources were screened and included. Additionally, the search strategy used across various databases will be detailed in a dedicated table, outlining the specific search terms, Boolean operators, and custom filters applied. By mapping the existing evidence, the review will likely identify the range of digital tools currently available, their key features, and the contexts in which they have been applied. It is anticipated that the findings will not only highlight effective strategies and common obstacles but also expose significant gaps in the literature. These insights will serve as a valuable foundation for designing a contextually appropriate mHealth programme tailored to the specific needs of health science students facing academic and emotional challenges.

## Discussion

This scoping review has several limitations. Firstly, it will only include studies published in English, potentially excluding relevant research in other languages. Secondly, the study would only look at research that was published between 2014 and 2024, which can leave out earlier but still relevant studies on stress management with mobile health interventions. Furthermore, the reliability and robustness of the results may be impacted by the scoping review methodology's failure to critically evaluate the caliber of the included research. The quality and accessibility of the data presented in the chosen studies may also have an impact on the review's conclusions, which could affect the synthesis's precision and comprehensiveness. Lastly, even though every attempt will be made to guarantee a thorough search strategy, which includes searching several databases and manually looking through review articles and meta-analyses, it is still possible that some pertinent research will be overlooked.

## Conclusion

This scoping review aims to synthesise literature on mobile health interventions for stress management among health science students in resource-limited universities. It seeks to map available solutions, assess their effectiveness, and explore contextual factors affecting adoption. The findings will guide policymakers, educators, healthcare professionals, and developers in addressing gaps and creating evidence-based, scalable digital mental health solutions tailored to local needs. Additionally, the review will inform institutional policies, advocate for integrating mobile interventions in wellness programs, and support further research and development of adaptable mental health applications.
